# Rearrangements of Cycloalkenyl Aryl Ethers

**DOI:** 10.3390/molecules21040503

**Published:** 2016-04-19

**Authors:** Mercedesz Törincsi, Melinda Nagy, Tamás Bihari, András Stirling, Pál Kolonits, Lajos Novak

**Affiliations:** 1 Department of Organic Chemistry and Technology, Budapest University of Technology and Economics, Gellért tér 4, Budapest 1111, Hungary; torincsi@mail.bme.hu (M.T.); nagymelynda@freemail.hu (M.N.); Kolonits@mail.bme.hu (P.K.); 2 Research Centre for Natural Sciences of the Hungarian Academy of Sciences, Department of Theoretical Chemistry, Magyar Tudósok Körútja 2, Budapest 1117, Hungary; bihari.tamas@ttk.mta.hu (T.B.); stirling.andras@ttk.mta.hu (A.S.)

**Keywords:** Claisen rearrangement, Cope rearrangement, phenol ethers, naphthyl ethers, reaction mechanism

## Abstract

Rearrangement reactions of cycloalkenyl phenol and naphthyl ethers and the acid-catalyzed cyclization of the resulting product were investigated. Claisen rearrangement afforded 2-substituted phenol and naphthol derivatives. Combined Claisen and Cope rearrangement resulted in the formation of 4-substituted phenol and naphthol derivatives. In the case of cycloocthylphenyl ether the consecutive Claisen and Cope rearrangements were followed by an alkyl migration. The mechanism of this novel rearrangement reaction is also discussed.

## 1. Introduction

Phenol and its derivatives play an important role in organic chemistry. They are highly useful building blocks in natural products and pharmaceuticals. They are also the starting compounds in the industrial synthesis of diverse antiseptics, dyes, aspirin, anilines, phenolic resins and caprolactams [1,2,3,4,5]. Furthermore, benzofurans, which can be prepared from phenols, have shown a wide range of biological activities. Substituted benzofuran (coumaran) is one of the most important heterocycles and is a powerful scaffold for many biological activities. Its derivatives have also been successfully applied in the synthesis of molecules which are pharmacologically active inhibitors against many diseases, viruses, microbes, fungi, and enzymes [6,7,8,9,10]. Naphthols and their methyl ethers also possess useful biologically activities. They show inhibitory activity with preferential inhibition on cyclooxygenase I and II [11,12].

Previously we developed methods for the preparation of cycloalkano-naphthofuran derivatives by the reaction of naphthol and cycloalka-1,3-dienes [13]. In those one-pot reactions we had assumed that the process went through the initial formation of naphthyl ether followed by acid catalyzed intramolecular cyclization. However, we were unable to isolate the naphthyl ether intermediates and under the forcing conditions some part of the product underwent fast dehydrogenation. With the aim of continuing our efforts to synthesize novel heterocyclic compounds, we have now examined the synthesis and rearrangement reaction of phenyl and naphthyl ethers. We report here the results and the preparation of the new compounds.

## 2. Results and Discussions

**Scheme 1:**
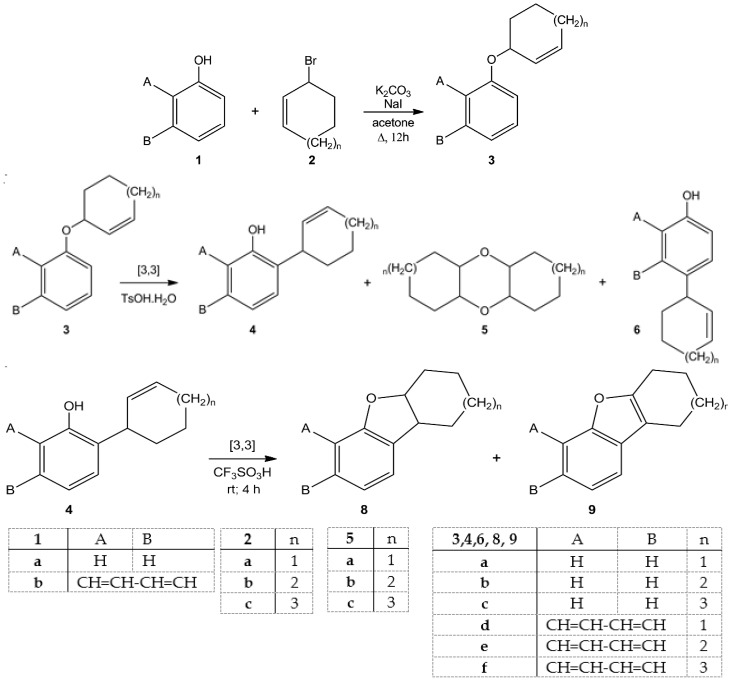
Acid-catalyzed rearrangement of ethers **3**.

Ethers **3a**–**f** were prepared by the reaction of compounds **1a** and **1b** and the appropriate bromocycloalk-1-ene **2** in acetone, in the presence of K_2_CO_3_ and NaI. These reactions yielded ethers **3** in good to acceptable yields. The attempted thermal reaction of ethers resulted only decomposition. TsOH·H_2_O (*p*Ka = −2.8)-catalyzed reactions of the phenol ethers **3a** and **3b** gave a mixture of three compounds (entries 1 and 2, Scheme 1). The major products were the 2-cycloalkyl derivatives **4a** and **4b**, formed by [3,3] rearrangement. The minor products were 4-substituted phenols **6a** and **6b** (Table 1). They were formed by consecutive Claisen- and Cope-rearrangements [14,15,16,17,18,19]. Under these strong acidic conditions, the decomposition of ethers **3a** and **4b** took place, and small amounts of dioxane derivatives **5a** and **5b** were also isolated.

**Scheme 2:**
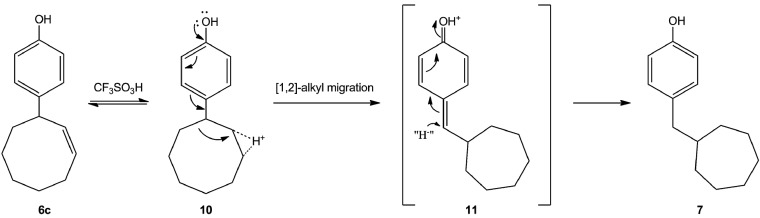
Ring-contraction rearrangement of compound **6c**.

Acid-catalyzed reaction of cyclooctylphenol **3c** yielded only Claisen product **4c**. However, treatment of **4c** with stronger acid, CF_3_SO_3_H (*p*K_a_ = −15), yielded an unexpected product **7**. A plausible reaction mechanism for the formation of this product is depicted in Scheme 2. In the Cope rearrangement of compound **4c** compound **6c** was formed. After protonation, a 1,2-alkyl shift took place on the conjugated acid **10** and then the quinoid intermediate took up a hydride anion [20,21,22].

We have investigated the generality of the above discussed reaction. Acid-catalyzed reaction of naphthyl ether **3d** also yielded three products (entry 4). Namely, the Claisen rearrangement product **4d**, the combined Claisen and Cope rearrangement product **6d**, and the dioxane derivative **5a**, in a 5:2:3 ratio. Essentially the same result was obtained with the cyclohepthyl ether homolog **3e** (entry 5). From this reaction compounds **4e**, **6e**, and the decomposition product **5b** were isolated.

The acid-catalyzed reaction of cyclooctylnaphthyl ether **3f** generated only one product, the *α-*substituted naphthol derivative **4f** (entry 6). Our effort to obtain the combined rearrangement product **6f** with stronger acid failed. Here, the first product **4f** showed a strong intramolecular cyclization tendency and the cycloocta[*b*]naphtho[2,1-*d*]furan derivative **8f** (*cis/trans* ring fusion ratio 1:2) was isolated. Later on we used CF_3_SO_3_H for the cyclization of compounds **4a**, **4b** and **4d**–**f**. The reactions were rather fast at room temperature and yielded predominantly compounds **8a**–**e**, besides trace amounts of compounds **9a**–**e**.

We also studied these cyclization reactions under microwave irradiation. The reactions were rather slow and to achieve complete cyclization we had to apply a higher temperature (200 °C) and longer reaction times. Under these conditions dehydrogenative aromatization took place and compounds **9** could be isolated.

**Figure 1:**
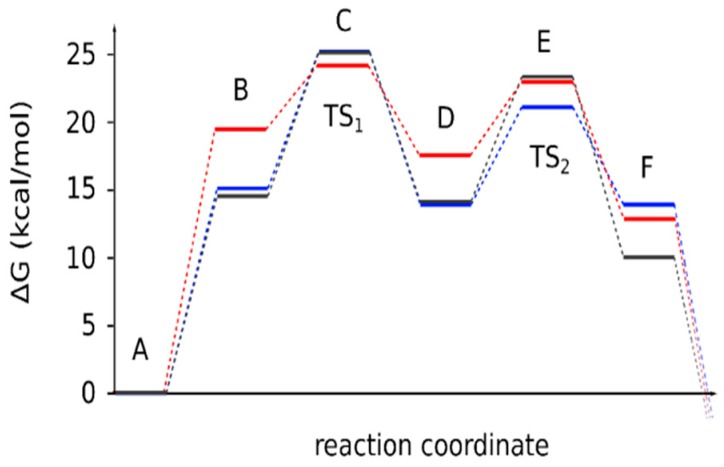
Free energy profiles for the Cope rearrangement reactions of compounds **4a**, **4b** and **4c**. The letters designate the reaction intermediate and transition states as displayed in Figure 2. The final deprotonation step is indicated by the last dashed lines. Color code: dark grey line—**4a**; blue—**4b**; red—**4c**.

**Figure 2:**
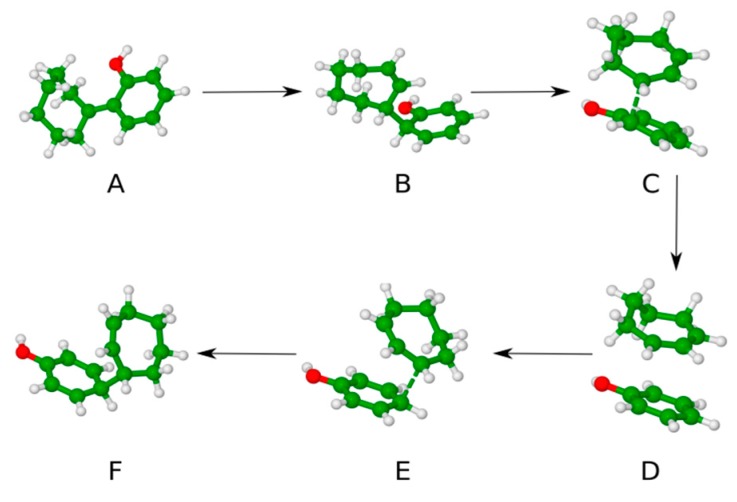
The mechanism of the proton-catalyzed Cope rearrangement. (**A**): the most stable protonated form; (**B**): suitable tautomeric form for Cope rearrangement; (**C**): first transition state (TS_1_); (**D**): intermediate structure; (**E**): second transition state (TS_2_); (**F**): protonated product state. Color code: green—carbon; red—oxygen; white—hydrogen.

**Figure 3:**
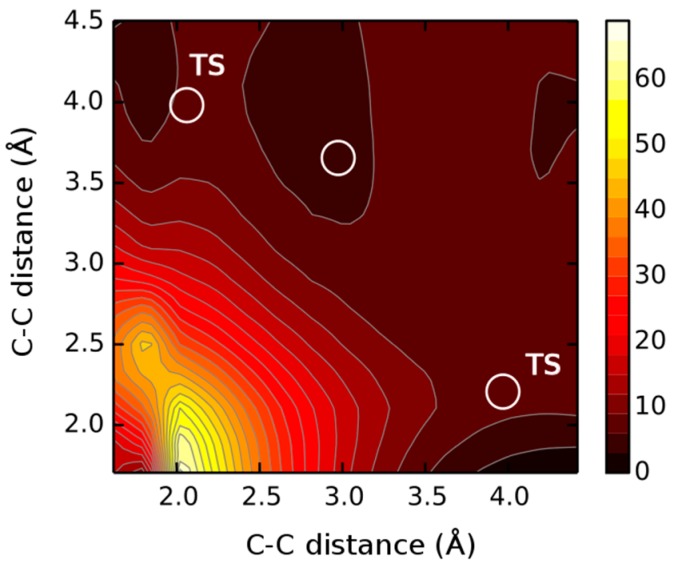
Calculated PES (in kcal/mol) for compound **4a** as a function of the breaking (horizontal axis) and forming CC bonds (vertical axis). The zero level is set to the most stable structure within the calculation domain. The most stable reactant and product regions are only partially shown at the upper left and lower right corners. TS and intermediate regions are indicated by white circles.

In order to understand this puzzling difference between the two activation modes we sought to understand the mechanism of the Cope rearrangement. To this end we have performed DFT calculations. First we have focused on the rearrangement of three molecules: 2-(2-cyclohexene-1-yl)-phenol (**4a**), 2-(2-cycloheptene-1-yl)-phenol (**4b**), and 2-(2-cyclooctene-1-yl)-phenol (**4c**). We have calculated both the thermal and the ionic (proton-catalyzed) routes. First we note that without proton catalysis the rearrangements hardly take place, because the bond breaking between the aromatic and aliphatic rings gives rise to a highly unstable intermediate (we have calculated more than 100 kcal/mol activation energies for this step). In contrast, proton catalysis allows a new route, where the bond breaking steps are facilitated by the excess protons. The calculated free energy profiles are shown in Figure 1, whereas the corresponding structures for the rearrangement of the cycloheptene ring are shown in Figure 2. To demonstrate why a two-step mechanism is preferred to a one-step rearrangement, a simple 2D potential energy surface (PES) scan has been performed as a function of the breaking and forming CC bonds. Figure 3 plots the calculated PES for compound **4a**. The PES shows that a hypothetical one-step process would cross a much higher energy region. Therefore the two-step process is favored. We have found entirely analogous routes for the other two molecules. The free energy values are listed in Table 2.

The most stable protonated form (A) features a benzylic structure which efficiently delocalizes the extra charge of the proton. The rearrangement implies however the presence of a proton at the C2 position on the aromatic ring which requires a tautomeric rearrangement and a 14–19 kcal/mol free energy investment. The next step is the bond breaking between the two rings and the formation of a peculiar intermediate state (D) where the positively charged aliphatic ring is stabilized by the close aromatic ring. This step requires 24–25 kcal/mol activation free energy which indicates that this process can indeed occur at ambient conditions. This is the rate-determining step of the process. In the following step the intermediate goes through a slightly lower barrier (21–23 kcal/mol range for the three compounds) and forms the protonated product (F). Comparison of the free energies of the product and reactant states in their neutral, unprotonated states reveals a moderate exergonicity for all the three rearrangements (between −1 and −6 kcal/mol). Assuming a few kcal/mol uncertainty for our computational methodology [23,24,25,26], we can conclude that the rearrangement reactions are reversible and an equilibrium of the two Cope-converted structures can be expected.

**Scheme 3:**
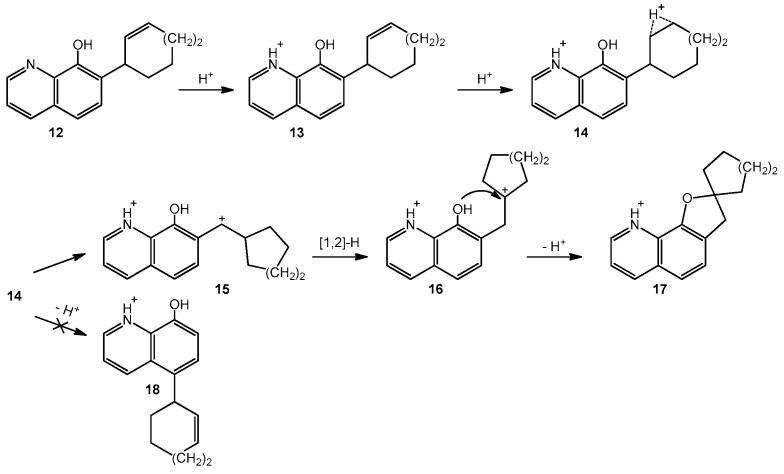
Rearrangement of quinoline **12**.

The stabilization effect of the aromatic ring in intermediate state D is crucial. We have found a compelling proof of this effect when explored a similar rearrangement paths for some analogous compounds: 7-(2-cycloalken-1-yl)-8-quinolines [27], where again 6-, 7- and 8-membered cycloalkene rings were considered. In an acidic environment the resting state of these compounds is the *N*-protonated form (Scheme 3). The rearrangement can then occur along two different pathways. In the first route additional protonation of the aromatic ring yields the proper doubly-protonated form for the Cope rearrangement. However, formation of the d-analogue intermediate would require the interaction of two positively charged rings, which is highly unfavorable and the system breaks apart. The second possibility is that the *N*-protonated form rearranges to the proper protonated form for the intramolecular Cope reaction where the 7C position of the quinoline is protonated. Due to the strong basicity of the *N* site, this is a much less favorable state by 29.0 kcal/mol free energy implying that this form cannot be observed in practice. The rate-determining step features a transition state at 42.0 kcal/mol on the free energy scale. These results indicate that the rearrangement of these quinolines cannot be observed under normal circumstances.

## 3. Experimental Section

### 3.1. General Information

Solvents were used as received from commercial vendors and no further attempts were made to purify or dry them. Mps were determined on a Büchi apparatus (Büchi labortechnik GmbH, Essen, Germany) and are uncorrected. Infrared (IR) spectra were recorded on an Alpha FT spectrophotometer (Bruker Bio spin GmbH, Ettlingen, Germany). ^1^H- and ^13^C-NMR spectra were obtained on a Bruker DRX-500 spectrometer. All NMR spectra are reported in ppm relative to TMS. MS spectra were conducted on a 6140 quadropole LC/MS instrument (Agilent, Santa Clara, CA, USA, ESI was +2 kV). Merck (Darmstadt, Germany) precoated silica gel 60 F_254_ plates were used for TLC and Kieselgel 60 for column chromatography. Solvent were mixed on a *v*/*v* basis. HPLC chromatographic analyses and separation were performed with a Waters 600 system (Waters GmbH, Eschborn, Germany) equipped with a photodiode array detector 990. The column used was a Supelcosil™ SPLC-18-DB, 250 mm × 10 mm. Microwave accelerated reactions were taken in a CEM Focused Microwave™ synthesis System (CEM Corporation, Matthews, NC, USA).

Phenol and naphth-1-ol were obtained from commercial sources and were the highest grade available. 3-Bromocyclohex-1-ene (**2a**) [28], 3-bromocyclohept-1-ene (**2b**) [29], and 3-bromocyclooct-1-ene (**2c**) [30] were prepared using the corresponding literature procedures.

The calculations have been performed by using the Gaussian 09 program package [20]. We have used density functional theory (DFT) with the dispersion-corrected, range-separated hybrid ωB97XD exchange-correlation functional [24]. Structural optimizations, transition state (TS) searches and vibrational calculations have been carried out using the 6-31+G * basis set. The Gibbs free energy contributions to the electronic energy have been calculated by employing the harmonic oscillator, rigid rotor, ideal gas approximation. The TS structures have been identified by having a single imaginary frequency. In addition mixed IRC-optimization calculations have been performed to confirm that a given TS connects the corresponding intermediate states. For the solvent effects we have applied implicit solvent model within the SMD formalism [25]. Since technical reasons do not allow to employ mixed solvents, water has been selected as a suitable choice to represent the strong acidic environment. For the final free energy profile the larger 6-311++G(3df, 3pd) basis set has been employed for a single point energy calculation (including the solvation effects) on the optimized structures, whereas the free energy contributions are taken from the calculations employing the smaller basis set.

### 3.2. Preparation of Ethers ***3***: General Procedure

To a cold stirred suspension of compound **1** (0.02 mol), K_2_CO_3_ (0.02 mol), and NaI (0.001 mol) in dry acetone (50 mL) was added bromocycloalkene **2** and the resulting mixture was refluxed for 12 h. After cooling to room temperature, the reaction mixture was quenched with water and then extracted with CH_2_Cl_2_ (2 × 50 mL). The combined organic layers were washed with water and dried (MgSO_4_). Evaporation of the solvent gave a residue which was purified by column chromatography on silica gel (20% EtOAc in hexane, as eluent).

*(2-Cyclohexen-1-yloxy)benzene* (**3a**) [31]. Yield: 65%: light yellow oil: *R_f_* = 0.77 (hexane–EtOAc 5:1). IR (KBr): 1599, 1584, 1492, 1445, 1298, 1288. 1235, 1077, 1065 cm^−1^. ^1^H-NMR (CDCl_3_): δ = 1.5–2.0 (m, 3H), 2.02 (m, 1H), 2.12 (m, 1H), 2.45 (m, 1H), C_4_’-C_6_’ H-s, 4.79 (br. s, 1H, C_1_’-H), 5.87 (m, 1H, C_2_’H), 5.96 (m, 1H, C_3_’H), 6.92 (m, 3H, C_2_-H, C_4_-H, and C_6_-H), 7.27 (t, *J* = 8 Hz, 2H, C_3_-H and C_5_-H). ^13^C-NMR (CDCl_3_): 19.03 (C-5’), 25.13 (C-4’), 28.32 (C-6’), 70.81 (C-1’), 115.89 (C-2 and C-6), 120.62 (C-4), 126.41 (C-3’), 129.47 (C-3 and C-5), 132.08 (C-2’), 157.84 (C-1). MS: *m/z* (%) = 174 (M^+^, 54), 145 (M^+^-C_2_H_5_, 74), 93 (M^+^-C_6_H_9_, 100).

*(2-Cyclohepten-1-yloxy)benzene* (**3b**) [32]. Yield: 68%: light yellow oil: *R_f_* = 0.72 (hexane–EtOAc 5:1). ^1^H-NMR (CDCl_3_): δ = 1.40 (m, 1H, C_5_’-H), 1.68 (m, 1H, C_6_’-H), 1.70 (C_7_’-H), 1.75 (m, 1H, C_5_’-H), 2.03 (m, 2H, C_6_’-H and C_7_’-H), 2.12 (m, 1H, C_4_’-H), 2.23 (m, 1H, C_4_’-H), 4.89 (m,1H, C_1_’-H), 5.82 (m, 1H, C_2_’-H), 5.85 (m, 1H, C_3_’-H), 6.87 (d, *J* = 8 Hz, 2H, C_2_-H and C_6_-H), 6.91 (t, *J* = 8 Hz, 1H, C_4_-H), 7.26 (*J* = 8 Hz, 2H, C_3_-H and C_5_-H). ^13^C-NMR (CDCl_3_): δ = 26.51 (C-5’), 27.49 (C-6’), 28.54 (C-4’), 33.10 (C-7’), 77.18 (C-1’), 115.65 (C-2 and (C-6), 120.47 (C-4), 129.41 (C-3 and C-5), 130.84 (C-3’), 135.74 (C-2’), 157.68 (C-1). MS: *m*/*z* (%) = 188 (M^+^, 58), 159 (M^+^-C_6_H_9_, 82), 93 (M^+^-C_7_H_11_, 100).

*(2-Cycloocten-1-yloxy)benzene* (**3c**) [33]. Yield: 74%: light yellow oil: *R_f_* = 0.77 (hexane–EtOAc 5:1). IR (KBr): 1598, 1584, 1492, 1452, 1299, 1238, 1171, 1038, 982 cm^−1^. ^1^H-NMR (CDCl_3_): δ = 1.4–1.8 (m, 7H), 2.08 (m, 1H), 2.20 (m, 1H), 2.28 (m, 1H, C_4_’-H), C_8_’ H-s, 5.08 (m, 1H, C_1_’-H), 5.52 (m, 1H, C_2_’-H), 5.73 (m, 1H, C_4_’-H), 6.87 (d, *J* = 8 Hz, 2H, C_2_-H and C_6_-H), 6.90 (d, *J* = 8 Hz, 1H, C_4_-H), 7.24 (t, *J* = 8 Hz, 2H, C_3_-H and C_5_-H). ^13^C-NMR (CDCl_3_): δ = 23.45 (C-7’), 26.22 (C-6’), 26.73 (C-4’), 29.07 (C-5’), 35.81 (C-8’), 75.03 (C-1’), 115.54 (C-2 and C-6), 120.41 (C-4), 129.32 (C-3 and C-5), 129.85 (C-3’), 133.16 (C-2’), 158.26 (C-1). MS: *m*/*z* (%) = 204 (M^+^, 46), 175 (M^+^-C_2_H_5_, 76), 95 (M^+^-C_8_H_13_, 100).

*1-(Cyclohex-2-enyloxy)naphthalene* (**3d**). Yield: 56%; yellow oil: *R_f_* = 0.84 (hexane–EtOAc 5:1). *R_t_* = 6.67 min. IR (KBr): 1598, 1586, 1491, 1448, 1432, 1396, 1235, 1035 cm^−1^. ^1^H-NMR (CDCl_3_): δ = 1.70 (m, 1H, C_5_’-H), 1.93 (m, 1H, C_5_’-H), 2.02 (m, 2H, C_6_’-H), 2.07 (m, 1H, C_4_’-H), 2.17 (m, 1H, C_4_’-H), 5.00 (m, 1H, C_1_’-H), 6.00 (m, 2H, C_2_’-H and C_3_’-H), 6.89 (d, *J* = 8 Hz, 1H, C_2_-H), 7.36 (t, *J* = 8 Hz, 1H, C_3_-H), 7.42 (m, 1H, C_6_-H), 7.78 (d, *J* = 8 Hz, 1H, C_5_-H), 8.29 (d, *J* = 8 Hz, 1H, C_8_-H). ^13^C-NMR (CDCl_3_): δ = 19.23 (C-5’9), 25.23 (C-4’), 28.51 (C-6’), 71.31 (C-1’), 106.36 (C-2), 120.06 (C-4), 122.45 (C-8), 125.00 (C-7), 125.83 (C-3), 126.28 (C-6), 126.42 (C-2’), 126.54 (C-8*a*), 127.40 (C-5), 132.10 (C-3’), 134.76 (C-4*a*), 153.66 (C-1). MS: *m*/*z* (%) = 224 (M^+^, 65), 195 (M^+^-C_2_H_5_, 52), 181 (M^+^-C_3_H_7_, 100). Anal. Calcd for C_16_H_16_O: C, 85.75; H, 7.14. Found: C, 85.52; H, 7.28.

*1-[(Z)-Cyclohept-2-enyloxy]naphthalene* (**3e**). Yield: 62%; yellow oil: *R_f_* = 0.9 (hexane–EtOAc 5:1). *R_t_* = 6.68 min. IR (KBr): 1577, 1506, 1460, 1444, 1395, 1265, 1236, 1154, 1095, 1061 cm^−1^. ^1^H-NMR (CDCl_3_): δ = 1.46 (m, 1H, C_5_’-H), 1.75 (m, 2H, C_5_’-H and C_6_’-H), 1.92 (m, 1H, C_7_’-H), 2.07 (C_6_’-H), 2.17 (m, 2H, C_4_’-H and C_7_’-H), 2.28 (m, 1H, C_4_’-H), 5.08 (d, *J*= 10 Hz, 1H, C_1_’-H), 5.90 (m, 2H, C_2_’-H and C_3_’-H), 6.76 (d, *J* = 8 Hz, 1H, C_2_-H), 7.34 (t, *J* = 8 Hz, 1H, C_3_-H), 7.38 (m, 1H, C_4_-H), 7.45 (m, 2H, C_6_-H and C_7_-H), 7.78 (d, *J* = 8 Hz, 1H, C_5_-H), 8.30 (d, *J* = 8 Hz, 1H, C_8_-H). ^13^C-NMR (CDCl_3_): δ = 26.62 (C-5’), 27.53 (C-6’), 28.58 (C-4’), 33.13 (C-7’), 77.56 (C-1’), 106.22 (C-2), 119.92 (C-4), 122.31 (C-8), 125.01 (C-7), 125.79 (C-3), 126.27 (C-8*a*), 127.41 (C-5), 131.04 (C-2’), 134.69 (C-4*a*), 135.73 (C-3’), 153.37 (C-1). MS: *m*/*z* (%) = 238 (M^+^, 15), 209 (M^+^-C_2_H_5_, 3), 181 (M^+^-C_4_H_9_, 13), 157 (M^+^-C_6_H_9_, 21), 144 (M^+^-C_7_H_10_, 100). Anal. Calcd for C_17_H_18_O: C, 85.72; H, 7.56. Found: C, 85.52; H, 7.38.

*1-[(Z)-Cyclooct-2-enyloxy]naphthalene* (**3f**). Yield: 58%; *R_f_* = 0.9 (hexane–EtOAc 5:1). IR (KBr): 1627, 1595, 1577, 1507, 1461, 1448, 1395, 1347, 1266, 1237, 1155, 1093, 1064 cm^−1^. ^1^H-NMR (CDCl_3_): *δ* = 1.54 (m, 2H, C_4_’-H and C_5_’-H), 1.72 (m, 4H, C_5_’-H, C_6_’-H and C_7_’-H), 1.90 (m, 1H, C_8_’-H), 2.22 (m, 2H, C_4_’-H and C_8_’-H), 2.35 (m, 1H, C_6_’-H), 5.28 (m, 1H, C_1_’-H), 5.60 (m, 1H, C_2_’-H), 5.78 (m, 1H, C_3_’-H), 6.78 (d, *J* = 8 Hz, 1H, C_2_-H), 7.31 (t, *J* = 8 Hz, 1H, C_3_-H), 7.39 (m, 1H, C_4_-H), 7.44 (m, 2H, C_6_-H and C_7_-H), 7.77 (m, 1H, C_5_-H), 8.30 (m, 1H, C_8_-H). ^13^C-NMR (CDCl_3_): δ = 23.45 (C-7’), 26.28 (C-6’), 26.76 (C-4’), 29.07 (C-5’), 35.80 (C-8’), 75.61 (C-1’), 106.34 (C-2), 119.87 (C-4), 122.26 (C-8), 124.94 (C-7), 125.84 (C-3), 126.14 (C-8*a*), 126.19 (C-6), 127.40 (C-5), 129.89 (C-3’), 133.22 (C-2’), 134.58 (C-4*a*), 153.93 (C-1). MS: *m*/*z* (%) = 252 (M^+^, 52), 209 (M^+^-C_3_H_7_, 8), 194 (M^+^-C_4_H_9_, 15), 181 (M^+^-C_5_H_11_, 169 (M^+^-C_6_H_11_, 28), 157 (M^+^-C_7_H_11_, 78), 144 (M^+^-C_8_H_12_, 100)), Anal. Calcd for C_18_H_20_O: C, 85.72; H, 7.93. Found: C, 85.54; H, 7.92.

### 3.3. Rearrangement of Ethers ***3***: General Procedure

A mixture of ether (2 mmol) and 3.8 g (2 mmol) PTS·H_2_O in 100 mL dry toluene was stirred and heated for the time indicated in Table 1. After cooling to room temperature water (50 mL) was added and the layers separated. The aqueous layer was extracted with toluene and the combined organic solutions were dried (MgSO_4_) and evaporated. The residue was purified by column chromatography (silica gel, hexane/EtOAc = 5:1).

*2-(2-Cyclohexen-1-yl)phenol* (**4a**) [34]. Yield: 25%; light yellow oil: *R_f_* = 0.63 (hexane–EtOAc 5:1). IR (KBr): 1598, 1491, 1432, 1288, 1235, 1172, 1035 cm^−1^. ^1^H-NMR (CDCl_3_): δ = 1.5-2.2 (m, 6H, 3CH_2_), 3.6 (m, 1H, CH), 5.85 (m, 1H, CH=), 6.08 (m, 1H, CH=), 6.74 (m, 1H, ArH), 6.85 (m, 1H, ArH), 7.14 (m, 2H, ArH). ^13^C-NMR (CDCl_3_): δ = 21.42, 25.01, 30.03, 37.99, 116.12, 120.61, 126.60, 128.01, 128.85, 129.56, 129.71, 153.60. MS: *m*/*z* (%) 174 (M^+^, 97), 159 (M^+^-15, 40), 145 (M^+^-C_2_H_5_, 100), 131 (M^+^-C_3_H_7_, 84).

*4-(2-Cyclohexen-1-yl)phenol* (**6a**) [35]. Yield: 36%; *R_f_* = 0.36 (hexane–EtOAc 5:1). *R_t_* = 4.48 min. IR (KBr): 3474 (OH), 1649, 1606, 1589, 1453, 1432, 1340, 1298, 1287, 1188 cm^−1^. ^1^H-NMR (CDCl_3_): δ = 1.67 (m, 3H, C_6_’-H and C_6_’-H), 2.12 (m, 3H, C_4_’-H and C_6_’-H), 4.12 (m, 1H, C_6_’-H), 5.45 (br.s, 1H, OH), 5.80 (m, 1H, C_2_’-H), 5.98 (m, 1H, C_3_’-H), 6.73 (d, *J* = 8 Hz, 1H, C_2_-H), 7.18 (d, *J* = 8 Hz, 1H, C_3_-H), 7.46 (m, 1H, C_7_-H), 7.50 (m, 1H, C_6_-H), 8.07 (d, *J* = 8 Hz, 1H, C_5_-H), 8.23 (d, *J* = 8 Hz, 1H, C_2_-H). ^13^C-NMR (CDCl_3_): δ = 20.72 (C-5’), 25.28 (C-4’), 30.92 (C-6’), 36.53 (C-1’), 108.05 (C-2), 122.41 (C-8), 123.43 (C-5), 124.69 (C-7), 124.89 (C-8*a*), 124.98 (C-3), 126.25 (C-6), 128.62 (C-3’), 130.45 (C-2’), 132.39 (C-4*a*), 134.30 (C-4). MS: *m*/*z* (%) = 174 (M^+^, 92%), 145 (M^+^-C_2_H_5_, 100%), 131 (M^+^-C_3_H_7_, 87%), 120 (M^+^-C_4_H_6_).

*Dodecahydrooxanthrene* (**5a**) [36]. Yield: 12%; *R_f_*= 0.9 (hexane–EtOAc 5:1). IR (KBr): 1458, 1445, 1432, 1358, 1267, 1256, 1195, 1177, 1160, 1116, 1048, 1032, 998 cm^−1^. ^1^H-NMR (CDCl_3_): δ = 1.52 (m, 4H, C_2_-H, C_3_-H, C_7_-H, C_8_-H), 1.81 (m, 4H, C_2_-H, C_3_-H, C_7_-H, C_8_-H), 1.89 (m, 4H, C_1_-H, C_4_-H, C_6_-H, C_9_-H), 2.46 (m, 4H, C_1_-H, C_4_-H, C_6_-H, C_9_-H), 4.45 (m, 4H, C_4*a*_-H, C_5*a*_-H, C_9*a*_-H, C_10*a*_-H). ^13^C-NMR (CDCl_3_): δ = 22.38 (C-2, C-3, C7, C-8), 31.97 (C-1, C-4, C-6, C-9), 55.20 (C-4*a*, C-5*a*, C-9*a*, C-10*a*).

*2-(2-Cyclohepten-1-yl)phenol* (**4b**) [31]. Yield: 32%; *R_f_* = 0.5 (hexane–EtOAc 5:1). IR (KBr): 3427 (OH), 1591, 1501, 1488, 1452, 1261, 1094, 1042 cm^−1^. ^1^H-NMR (CDCl_3_): δ = 1.48 (m, 1H, C_5’_-H), 1.66 (m, 1H, C_6’_-H), 1.82 (m, 1H. C_5’_-H), 1.85 (m, 2H, C_7_’-H), 1.98 (m, 1H, C_6’_-H), 2.24 (m, 1H, C_4’_-H), 2.32 (m, 1H, C_4’_-H), 3.73 (m, 1H, C_1’_-H), 5.75 (m, 1H, C_2’_-H), 5.94 (C_3’_-H), 6.79 (d, *J*= 8 Hz, 1H, C_6_-H), 6.89 (d, *J* = 8 Hz, 1H, C_4_-H), 7.10 (td, *J* = 8 Hz and 1.5 Hz, 1H. C_5_-H), 7.16 (dd, *J* = 8 Hz and 1.5 Hz, 1H. C_3_-H). ^13^C-NMR (CDCl_3_): δ = 27.16 (C-5’), 28.86 (C-4’), 29.97 (C-6’), 34.14 (C-7’), 41.84 (C-1’), 115.91 (C-6), 120.87 (C-4), 127.22 (C-5), 128.47 (C-3), 132.91 (C-2), 133.39 (C-3’), 135.49 (C-2’), 153.00 (C-1). MS: *m*/*z* (%) = 188 (M^+^, 64), 187 (M^+^-H, 100), 173 (M^+^-CH_3_, 36), 159 (M^+^-C_2_H_5_, 92).

*2-(2-Cyclooct-1-yl)phenol* (**4c**) [35]. Yield: 47%; *R_f_* = 0.76 (hexane–EtOAc 5:1). IR (KBr): 3428 (OH), 1590, 1501, 1487, 1452, 1235, 1195, 1043 cm^−1^. ^1^H-NMR (CDCl_3_): δ = 1.4–2.5 (m, 10 H, 5CH_2_), 3.9 (m, 1H, CH), 5.20 (m, 1H. OH), 5.50 (m, 1H, CH=), 5.93 (m, 1H, CH=), 6.78 (m, 2H, ArH), 6.93 (m, 1H, ArH), 7.12 (m, 1H, ArH), 7.22 (m, 1H, ArH). ^13^C-NMR (CDCl_3_): δ = 25.62, 26.57, 26.71, 29.65, 33.79, 35.31, 115.92, 120.81, 126.51, 127.34, 131.15, 132.30, 132.89, 154.14. MS: *m*/*z* (%) = 202 (M^+^, 48), 201 (M^+^-H, 100), 187 (M^+^-CH_3_, 42), 173 (M^+^-C_2_H_5_, 87).

*2-(Cyclohex-2-enyl)naphthalen-1-ol* (**4d**) [36,37]. Yield: 37%; light yellow oil; *R_f_* = 0.78 (hexane–EtOAc 5:1); *R_t_* = 7.18 min. IR (KBr): 3454 (OH), 3053, 3016, 1654, 1649, 1599, 1572, 1508, 1465, 1444, 1386, 1358, 1269, 1177, 1131, 1057 cm^−1^. ^1^H-NMR (CDCl_3_): δ = 1.70 (m, 1H, C_5_’-H), 1.77 (m, 1H, C_6_’-H), 1.88 (m, 1H, C_5_’-H), 2.06 (m, 1H, C_6_’-H), 2.20 (m, 2H, C_4_’-H), 3.60 (m, 1H, C_1_’-H), 6.00 (m, 1H, C_2_’-H), 6.17 (m, 1H, C_3_’-H), 6.19 (s, 1H, OH), 7.20 (d, *J* = 8.3 Hz, 1H, C_3_-H), 7.36 (d, *J* = 8.3 Hz, 1H, C_4_-H), 7.42 (m, 1H, C_6_-H), 7.44 (m, 1H, C_7_-H), 7.75 (m, 1H, C_5_-H), 8.17 (m, 1H, C_8_-H). ^13^C-NMR (CDCl_3_): δ = 21.76 (C-5’), 25.07 (C-4’), 30.14 (C-6’), 39.76 (C-1’), 119.95 (C-4), 121.54 (C-8), 123.56 (C-2), 125.14 (C-7), 125.17 (C-8*a*), 125.68 (C-6), 127.37 (C-5), 128.02 (C-3), 129.89 (C-2’), 132.16 (C-3’), 133.47 (C-4*a*), 149.42 (C-1). MS: *m*/*z* = 224 (M^+^, 100), 195 (M^+^-C_2_H_5_, 75), 165 (M^+^-C_4_H_11_, 43), 152 (34), 139 (22).

*2-[(Z)-Cyclohept-2-enyl]naphthalen-1-ol* (**4e**).Yield: 36%; *R_f_* = 0.8 (hexane–EtOAc 5:1); *R_t_* = 7.49 min. IR (KBr): 3442 (OH), 3053, 3013, 1654, 1649, 1599, 1572, 1508, 1442, 1385, 1356, 1290, 1267, 1230, 1177, 1112, 1070, 1021 cm^−1^. ^1^H-NMR (CDCl_3_): δ = 1.55 (m, 1H, C_5_’-H),1.60 (m, 1H, C_6_’-H), 1.70 (m, 1H, C_6_’-H), 1.95 (m, 2H, C_5_’-H), C_6_’-H)), 1.99 (m, 1H, C_7_’-H), 2.04 (m, 1H, C_7_’-H), 2.33 m, 1H, C_4_’-H), 2.43 (m, 1H, C_4_’-H), 4.65 (m, 1H, C_1_’-H), 5.83 (m, 1H, C_2_’-H), 5.86 (s, 1H, OH), 6.01 (m, 1H, C_3_’-H), 7.38 (d, *J* = 8 Hz, 1H, C_3_-H), 7.43 (m, 3H, C_4_-H), 7.76 (dd, *J* = 7.5 and 1.5 Hz, C_5_-H), 8.18 (dd, *J* = 7 and 2 Hz, C_8_-H). ^13^C-NMR (CDCl_3_): δ = 27.25 (C-6’), 28.90 (C-6’), 29.53 (C-4’), 29.61 (C-5’), 33.59 (C-7’), 43.27 (C-1’), 120.120.32 (C-4), 121.42 (C-8), 125.02 (C-2), 125.62 (C-3), 125.67 (C-8*a*), 126.76 (C-7), 127.40 (C-6), 127.47 (C-5), 133.30 (C-3’), 134.66 (C-2’), 134.76 (C-4*a*), 148.15 (C-1). MS: *m*/*z* (%) = 238 (M^+^, 100), 221 (M^+^-OH, 7.6), 209 (M^+^-C_2_H_5_, 23), 195 (M^+^-C_3_H_7_, 55). Anal. Calcd for C_17_H_18_O: C, 85.67; H, 7.61. Found: C, 85.92; H, 7.42.

*Dodecahydro-1H,6aH-dicyclohepta[b,e][1,4]dioxine* (**5b**). Yield: 16%; *R_f_* = 0.90 (hexane–EtOAc 5:1). *R_t_* = 5.62 min. IR (KBr): 1577, 1453, 1428, 1400, 1266, 1235, 1216, 1160, 1118, 1094, 1066, 1003 cm^−1^. ^1^H-NMR (CDCl_3_): δ = 1.66 (m, 8H, C_2_-H, C_3_-H, C_4_-H, C_8_-H, C_9_-H, and C_10_-H), 1.87 (m, 4H, C_2_-H, C_4_-H, C_8_-H, and C_10_-H, 2.07 (m, 4H, C_1_-H, C_5_-H, C_7_-H, and C_11_-H), 2.35 (m, 4H, C_1_-H, C_5_-H, C_7_-H, and C_11_-H), 4.65 (m, 4H, C_5*a*_-H, C_6*a*_-H, C_11*a*_-H, and C_12*a*_-H). ^13^C-NMR (CDCl_3_): δ = 23.18 (C-2, C-4, C-8, and C-10), 26.45 (C-3 and C-9), 33.21 (C-1, C-5, C-7 and C-11), 60.16 (C-5*a*, 6*a*, 11*a* and 12*a*). MS: *m*/*z* (%) = 224 (M^+^, 68), 183 (M^+^-C_3_H_5_), 165 (M^+^-C_3_H_5_O, 92), 155 (M^+^-C_5_H_9_, 100). Anal. Calcd for C_14_H_24_O_2_: C, 75.51; H, 10.70. Found: C, 75.78; H, 10.89.

*2-[(Z)-Cyclooct-2-enyl]naphthalen-1-ol* (**4f**). Yield: 52%; *R_f_* = 0.83 (hexane–EtOAc 5:1). ^1^H-NMR (CDCl_3_): δ = 1.45 (m, 1H, C_5_’-H),1.53 (m, 1H, C_6_’-H), 1.68 (m, 1H, C_7_’-H), 1.83 (m, 3H, C_5_’-H, C_6_’-H, C_7_’-H), 1.97 (m, 1H, C_8_’-H), 2.02 (m, 1H, C_8_’-H), 2.31 (m, 1H, C_4_’-H), 2.54 (m, 1H, C_4_’-H), 4.07 (m, 1H, C_1_’-H), 5.53 (m, 1H, C_2_’-H), 5.79 (s, 1H, OH), 6.00 (m, 1H, C_3_’-H), 7.37 (d, *J* = 8 Hz, 1H, C_3_-H), 7.44 (m, 3H, C_4_-H), 7.76 (dd, *J* = 7.5 and 1.5 Hz, C_5_-H), 8.18 (dd, *J* = 7 and 2 Hz, C_8_-H). ^13^C-NMR (CDCl_3_): δ = 25.64 (C-7’), 26.48 (C-6’), 26.80 (C-4’), 29.61 (C-5’), 33.59 (C-8’), 35.59 (C-1’), 120.13 (C-4), 121.54 (C-8), 124.02 (C-2), 124.17 (C-3), 124.76 (C-8*a*), 125.17 (C-7), 125.63 (C-6), 127.37 (C-5), 132.80 (C-3’), 132.88 (C-2’), 133.25 (C-4*a*), 149.26 (C-1). MS: *m*/*z* (%) = 252 (M^+^, 100), 251 (M^+^-H, 69), 223 (M^+^-C_2_H_5_, 60). Anal. Calcd for C_18_H_20_O: C, 75.72; H, 7.93. Found: C, 75.79; H, 8.14.

*4-(Cyclohex-2-enyl)naphthalen-1-ol* (**6d**). Yield: 28%; *R_f_* = 0.5 (hexane–EtOAc 5:1). *R_t_* = 7.02 min. IR (KBr): 3331 (OH), 3058, 3018, 1626, 1599, 1587, 1517, 1445, 1377, 1354, 1302, 1264, 1212, 1146, 1049 cm^−1^. ^1^H-NMR (CDCl_3_): δ = 1.67 (m, 3H, C_5_’-H and C_6_’-H), 2.12 (m, 3H, C_4_’-H and C_6_’-H), 4.12 (m, 1H, C_1_’-H), 5.45 (br.s, 1H, OH), 5.80 (m, 1H, C_2_’-H), 5.98 (m, 1H, C_3_’-H), 6.73 (d, *J* = 8 Hz, 1H, C_2_-H), 7.18 (d, *J* = 8 Hz, 1H, C_3_-H), 7.46 (m, 1H, C_7_-H), 7.50 (m, 1H, C_6_-H), 8.07 (d, *J* = 8 Hz, 1H, C_5_-H), 8.23 (d, *J* = 8 Hz, 1H, C_8_-H). ^13^C-NMR (CDCl_3_): δ = 20.72 (C-5’), 25.28 (C-4’), 30.92 (C-6’), 36.53 (C-1’), 108.05 (C-2), 122.41 (C-8), 123.43 (C-5), 124.69 (C-7), 124.89 (C-8*a*), 124.98 (C-3), 126.25 (C-6), 128.62 (C-3’), 130.45 (C-2’), 132.39 (C-4*a*), 134.30 (C-4), 149.96 (C-1). MS: *m*/*z* (%) = 224 (M^+^, 100), 207 (M^+^-OH, 6), 195 (M^+^-C_2_H_5_, 74), 181 (M^+^-C_3_H_7_, 70), 165 (39), 152 (36). Anal. Calcd for C_16_H_16_O: C, 85.68; H, 7.19. Found: C, 85.43; H, 7.39.

*4-(Cyclohept-2-enyl)naphthalen-1-ol* (**6e**). Yield: 32%; *R_f_* = 0.42 (hexane–EtOAc 5:1). *R_t_* = 7.47 min. IR (KBr): 3357 (OH), 3072, 3052, 1626, 1601, 1589, 1516, 1443, 1378, 1355, 1275, 1258, 1215, 1146, 1054, 1014 cm^−1^. ^1^H-NMR (CDCl_3_): δ = 1.53 (m, 1H, C_5_’-H), 1.74 (m, 1H, C_6_’-H), 1.85 (m, 1H, C_5_’-H), 1.94 (m, 2H, C_7_’-H), 1.99 (m, 1H, C_6_’-H), 2.32 (m, 2H, C_4_’-H), 4.1 (br.s, 1H, OH), 4.20 (m, 1H, C_1_’-H), 5.80 (m, 1H, C_2_’-H), 5.88 (m, 1H, C_3_’-H), 6.76 (d, *J* = 8 Hz, 1H, C_2_-H), 7.23 (d, *J* = 8 Hz, 1H, C_3_-H), 7.47 (m, 1H, C_7_-H), 7.51 (m, 1H, C_6_-H), 8.04 (d, *J* = 8.5 Hz, 1H, C_5_-H), 8.23 (d, *J* = 8.5 Hz, 1H, C_8_-H). ^13^C-NMR (CDCl_3_): δ = 27.17 (C-5’), 28.88 (C-4’), 30.64 (C-6’), 35.01 (C-7’), 42.35 (C-1’), 108.16 (C-2), 122.30 (C-8), 123.82 (C-3), 123.92 (C-5), 124.72 (C-7), 124.88 (C-8*a*), 126.15 (C-7), 131.28 (C-3’), 132.14 (C-4*a*), 136.09 (C-4), 137.75 (C-2’), 149.79 (C-1). MS: *m/z* (%) = 238 (M^+^, 100), 221 (M^+^-OH, 10), 209 (M^+^-C_2_H_5_, 32), 195 (M^+^-C_3_H_7_, 181 (M^+^-C_4_H_9_, 75), 169 (M^+^-C_5_H_9_, 45). Anal. Calcd for C_17_H_18_O: C, 85.67; H, 7.61. Found: C, 85.78; H, 7.89.

*4-(Cycloheptylmethyl)phenol* (**7**). A mixture of **4f** (192 mg, 0.95 mmol) and CF_3_SO_3_H (1.6 mL, 1.9 mmol) was stirred under argon at room temperature for 1 h. The reaction mixture was quenched with 1 N NaOH (3 mL) and then extracted with chloroform (3 × 10 mL). The combined organic layers were dried (MgSO_4_) and concentrated *in vacuo*. The residue was purified by column chromatography to yield **7** (60 mg, 32%), light yellow oil. *R_f_* = 0.6 (hexane–EtOAc 5:1). *R_t_* = 7.54 min. IR (KBr): 3363 (OH), 1612, 1597, 1513, 1458, 1445, 1375, 1359, 1223, 1171, 1043 cm^−1^. ^1^H-NMR (CDCl_3_): δ = 1.15 (m, 2H, C_2_’-H and C_7_’-H), 1.37 (m, 2H, C_3_’-H and C_6_’-H), 1.49 (m, 2H, C_4_’-H and C_5_’-H), 1.60 (m, 4H, C_3_’-H, C_4_’-H, C_5_’-H and C_6_’-H), 1.68 (m, 3H, C_1_’-H, C_2_’-H and C_7_’-H), 2.43 (d, *J* = 7 Hz, 2H, CH_2_), 4.7 (br.s, 1H, OH), 6.73 (d, *J* = 8 Hz, 2H, C_2_-H and C_6_-H), 7.00 (d, *J* = 8 Hz, 2H, C_3_-H and C_5_-H). ^13^C-NMR (CDCl_3_): δ = 26-36 (C-3’ and C-6’), 28.40 (C-4’ and C-5’), 34.32 (C-2’ and C-7’). 41.50 (C-1’), 43.59 (CH_2_), 114.90 (C-2 and C6), 130.21 (C-3 and C-5), 134.11 (C-4), 153.41 (C-1). MS: *m*/*z* (%) = 204 (M^+^, 16), 203 (M^+^-1, 100). Anal. Calcd for C_14_H_20_O: C, 82.30; H, 9.87. Found: C, 82.58; H, 9.69.

### 3.4. General Procedures for Cyclization of Compounds ***4a***,***b*** and ***4d**–**f***

Method A: A mixture of **4** (1 mmol) and 0.30 g (0.18 mL, 2 mmol) of CF_3_SO_3_H was stirred at room temperature for 4 h. The reaction mixture was quenched with water (15 mL), the resulting solution was basified with 1 N NaOH solution and extracted with CH_2_Cl_2_ (3 × 15 mL). The combined organic layers were dried over MgSO_4_; solvent was evaporated in vacuo and the residue was purified by column chromatography.

Method B: Compounds **4** (3 mmol) were heated in microwave reactor at 210 °C for 8 h. After cooling, the reaction mixture was purified by column chromatography.

*1,2,3,4,4a,9b-Hexahydrodibenzo[b,d]furan* (**8a**) [38]. Method A. Yield 52%.

*1,2,3,4-Tetrahydrodibenzo[b,d]furan* (**9a**) [39]. Method B. Yield 46%.

*6,7,8,9,10,10a-Hexahydro-5aH-benzo[b]cyclohepta[d]furan* (**8b**) [39]. Method B. Yield: 14%.

*7,8,9,10-Tetrahydro-6H-benzo[b]cyclohepta[d]furan* (**9b**) [39]. Method B. Yield 56%.

*5a,6,7,8,9,10,11,11a-Octahydrobenzo[b]cycloocta[d]furan* (**8b**) [40]. Method A. Yield: 56%.

*6b,7,8,9,10,10a-Hexahydrobenzo[b]naphtho[2,1-d]furan* (**8d**). Method A. Yield: 45%, light yellow oil. *R_f_* = 0.76 (hexane#x2013;EtOAc 5:1). *R_t_* = 6.23 min. IR (KBr): 1637, 1572, 1508, 1441, 1374, 1351, 1258, 1176, 1074, 1039 cm^−1^. ^1^H-NMR (CDCl_3_): δ = 1.95 (m, 2H, C_8_-H), 1.97 (m, 2H, C_9_-H), 270 (m, 2H, C_7_-H), 2.82 (m, 2H, C_10_-H), 4.87 (m, 1H, C_6b_-H), 4.89 (m, 1H, C_10a_-H), 7.27 (t, *J* = 8 Hz, 1H, C_3_-H), 7.40 (d, *J* = 8 Hz, 1H, C_6_-H), 7.44 (t, *J* = 8 Hz, 1H, C_2_-H), 7.55 (d, *J* = 8 Hz, 1H, C_5_-H), 7.80 (d, *J* = 8 Hz, 1H, C_4_-H), 8.20 (d, *J* = 8 Hz, 1H, C_1_-H). ^13^C-NMR (CDCl_3_): δ = 21.80 (C-7), 22.90 (C-8), 23.07 (C-9), 23.61 (C-10), 44.70 (C-10), 89.50 (C-6b), 117.75 (C-1), 118.55 (C-11b), 121.35 (C-5), 124.05 (C-3), 125.98 (C-2), 128.34 (C-4), 130.87 (C-4a), 149.35 (C-11a). MS: *m/z* (%) = 224 (M^+^, 64), 195 (M^+^-C_2_H_5_, 7.5), 181 (M^+^-C_3_H_7_, 42), 170 ((M^+^-C_4_H_6_, 100), 152 (M^+^-C_4_H_8_O, 15). Anal. Calcd for C_16_H_16_O: C, 85.72; H, 7.12. Found: C, 85.58; H, 7.59.

*7,8,9,10-Tetrahydrobenzo[b]naphtho[2,1-d]furan.* (**9d**) [13]. Method B. Yield 53%.

*7,8,9,10,11,11a-Hexahydro-6bH-cyclohepta[b]naphtho[2,1-d]furan* (**8e**) [13]. Method A. Yield: 49%.

*7,8,9,10,11-Pentahydrocyclohepta[b]naphtho[2,1-d]furan* (**9e**) [13]. Method B. Yield: 67%.

*6b,7,8,9,10,11,12,12a-Octahydrocycloocta[b]naphtho[2,1-d]furan* (**8f**) [13]. Method B. Yield: 48%.

## 4. Conclusions

The acid-catalyzed Claisen and Cope rearrangements of cycloalkenyl phenyl and naphthyl ethers **3** afforded 2- and 4-substituted phenols and naphth-1-ols **4** and **6**, respectively, under moderate temperature conditions. The acid-catalyzed intramolecular cyclization of compounds **4** gave the corresponding cycloalkanobenzofuran and cycloalkanonaphthofurans **8a**–**c** and **8e**,**f**. In the microwave assisted reactions of compounds **4** partial aromatization of the products **8** took place and compounds **9** were isolated as a result of the required higher reaction temperature. The divergent product formations of the acidic and thermal activations have been rationalized by DFT calculations.

The acid-catalyzed reaction of 2-cyclooctanylphenol **4f** represents an unexpected twist. After the Cope rearrangement, a migration of the alkyl group took place and as a result of this ring contraction reaction we could isolate 4-cyclohepthylmethylphenol (**7**).

## Figures and Tables

**Table 1:** Acid-catalyzed reaction of ethers **3a**–**f**.

Entry	Starting Materials	Temperature (°C)	Reaction Time (h)	Product(s) (Yields %)
1	**3a**	20	24	**4a** (28); **6a** (22); **5a** (12)
2	**3b**	50	12	**4b** (26); **6b** (20); **5b** (14)
3	**3c**	50	22	**4c** (47)
4	**3d**	20	24	**4d** (37); **6d** (14); **5a** (14)
5	**3e**	20	40	**4e** (22); **6e** (15); 5b (16)
6	**3f**	50	8	**4f** (47)

**Table 2:** Relative free energies of the reactant, product and intermediate states and the reaction free energies (in neutral form, ΔG_r_) in kcal/mol for compounds **4a**, **4b** and **4c**.

Reaction Stages	4a	4b	4c
A	0	0	0
B	14.5	15.1	19.4
C (TS_1_)	25.1	25.2	24.1
D	14.1	13.9	17.5
E (TS_2_)	23.2	21.1	22.9
F	10.0	13.9	12.8
ΔG_r_	−0.9	−1.5	−5.6
